# Surgery.—Hernia

**Published:** 1905-01-07

**Authors:** 


### PROGRESS IN MEDICINE AND SURGERY.

### SURGERY.—HERNIA.

Hernia in Children.—Edred Corner,[1] in a postgraduate lecture on this subject, points out that the two chief factors in the production of hernia in children are a predisposing one, the congenital sac, and an exciting one, gastro-intestinal fermentation with the production of gas, abdominal distension, and increase of intra-abdominal tension. Many patients with a congenital sac do not develop a hernia until adult life, so that it would appear that the second, or exciting, cause was the more active factor in producing the hernia. Also, according to the author, many of the hernial sacs in young children are acquired, and he lays great stress on the importance of dietetics for the prevention of this gastro-intestinal disturbance, the exciting cause, in his opinion, of most of the herniæ of children. The question of the connection between phimosis and hernia is discussed, and the view put forward that in most cases the opening in the prepuce is not small enough to cause actual obstruction to the flow of urine, and that if it were so the prepuce would become ballooned during the act of micturition which is not observed by the mothers; that sometimes the narrowed meatus is the real cause of obstruction, and that many foreskins are unnecessarily removed and no benefit to the hernia follows. He argues that the hernia is caused by the continuance or persistence of the increase of intra-abdominal pressure as arising from gastro-intestinal fermentation rather than by sudden and violent increment of pressure which would arise from muscular efforts and strainings. In adults the conditions would appear to be reversed. The value of trusses in the treatment is next considered. It is assumed that when cure occurs it is by causing matting of the tissues about the neck of the sac, and perhaps in a few cases by obliteration of the neck of the sac. The skein and ordinary girdle trusses are referred to, the former as inefficient, though of some use, and the latter as likely to cause atrophy of the testes from pressure on the structures of the cord. The suggestion is made that those hernias which are cured by application of a truss have acquired sacs, and that the congenital ones are only cured by operation; and, further, that as the intra-abdominal distension ceases and growth continues, there is a tendency for the sac of an acquired hernia to be retracted into the abdomen, and in this way it may be pulled right up so that the hernia ceases to exist. A similar thing may happen to some extent in the case of the congenital sacs, accounting for the late appearance of imperfect descent of the testis. If the hernia is allowed to exist many years uncured in the child, it is apt to lead to an enlarged circular internal ring, which in the adult may be very difficult of closure. With regard to treatment, infants up to four or five years of age should be treated by dieting, rest in bed, and a light truss except when the hernia is large and uncontrollable, irreducible, or strangulated; at four to five years old they should be operated on. This age is selected because then the internal ring will grow properly afterwards; the operation is easier than at an earlier age, and the prognosis of the operation is very good. Umbilical and ventral hernia of children are considered to be caused by persisting intra-abdominal tension as mentioned above.

**Radical Cure of Hernia.**—Kammerer,[2] after briefly reviewing the methods that have been adopted for the radical cure of *femoral* hernia, thinks the most satisfactory is one of those having a transverse incision above Poupart's ligament. The following details of Lotheissen's operation are given:—(1) An incision parallel to Poupart's ligament and a little above the same, dividing the fibres of the external oblique. This incision extends into the external inguinal ring. (2) Exposure of the neck of the sac by entering between Poupart's ligament and the internal oblique muscle. (3) Dislocation of the sac by pulling the same, if small, into the opening above Poupart's ligament. (4) In large hernia, dissection of the skin at the lower edge of the original incision, exposure of the external surface of the sac, incision and reduction of its contents into the abdominal cavity, deligation and removal of the sac, and finally dislocation of the stump of the sac in the manner previously described for small hernia. (5) Suture of the edge of the transversalis and internal oblique muscles to Cooper's ligament. (6) Sutures of the incisions in the aponeurosis and the skin separately. In the case of very large or irreducible hernias it may be necessary to expose the sac on the crural aspect as well, and in strangulated hernias to divide Poupart's ligament; but this should be done from without inwards. The incision above Poupart's ligament as described gives much better access to the neck of the sac than the crural one, and there is far more room for such manipulations as resection of the gut, should this be necessary, as in the case of strangulated ruptures. Moynihan[3] points out the unsatisfactory results from operations on *umbilical* hernias; he briefly mentions the methods described in works on operative surgery and various points in the evolution of the radical operation, and finally favours most one of the overlapping methods, the latest—that of W. J. Mayo—being described in full. The overlapping is from above downwards. The toilet of the skin is of the greatest importance. The hernia is marked off by two curved incisions (placed transversely to the long axis of body), each about six or eight inches long, meeting at their extremities. These incisions are deepened until the anterior sheath of the rectus, a little distance from the neck of the sac, is exposed. By gauze-stripping the sheath is exposed and cleaned for an area about five by four inches around the neck of the sac. By this time the whole hernial mass is freed from its surroundings and is attached only by its stalk at the umbilicus. The neck of the sac is carefully opened and enlarged by cutting through the peritoneum in a circular manner. The contents are examined, and if only omentum be found in the pedicle it is ligatured in several places and removed and the stump reduced. Two transverse incisions are now made from the margins of the hernial orifice through all the structures of the abdominal wall for about $1\frac{1}{2}$ inch on either side. From the under surface of the upper flap the peritoneum is stripped up for about 1 inch to $1\frac{1}{2}$ inch, so that a pocket is formed between the peritoneum behind and the aponeurotic structures in front. The upper flap is sutured above the lower in the following manner:—A series of mattrass sutures is passed, the needle being introduced through the upper flap about 2 inches from the edge, and then transversely through the lower flap, about 1 inch from the edge, and finally pierces from behind forwards the upper flap about half an inch from the original point of entry. All the sutures are passed before any are tied. When all are in place they are gently pulled on and the lower flap is adjusted in the pocket made by stripping up the peritoneum. The sutures are tied and cut and the edge of the upper flap stitched by a continuous catgut suture to the anterior sheath of the rectus and the wound closed. Kangaroo tendon is recommended for the mattrass sutures. Steffen[4] gives his results of the alcohol injection treatment of reducible hernias. Schwalbe's original procedure consisted in the injection into the tissues around the neck of the sac of two to three grammes of 20 to 70 per cent. alcohol, the underlying principle being that a connective tissue hyperphasia should follow such an irritation. The injections were made daily for four to 14 days. Steffen's modification is that he uses 100 grammes of 50 per cent. alcohol with 10 drops each of dilute phosphoric acid and formalin; and if this fails, he substitutes two or three grains of extract of oak-bark. The longest treatment was four years, the shortest one year. The following analysis of 1,182 hernias of all sorts is given with the following results:—75·3 per cent. cured, 7·1 improved, and 17·6 not cured; 12·5 per cent. relapsed. The author does not say that he prefers this method to the radical operation, but remarks that there are many patients who will not submit to that procedure, and for them the injection treatment may be used with good success.

[1] Lancet, Aug. 20, 1904. [2] Annals of Surg., June 1904. [3] Lancet, July 3, 1904. [4] Med. Rec., June 18, 1904.

*(To be continued.)*

